# The Potential Use of Forensic DNA Methods Applied to Sand Fly Blood Meal Analysis to Identify the Infection Reservoirs of Anthroponotic Visceral Leishmaniasis

**DOI:** 10.1371/journal.pntd.0004706

**Published:** 2016-05-18

**Authors:** Ehud Inbar, Philip Lawyer, David Sacks, Daniele Podini

**Affiliations:** 1 Laboratory of Parasitic Diseases, National Institute of Allergy and Infectious Diseases, National Institutes of Health, Bethesda, Maryland, United States of America; 2 The Department of Forensic Sciences, Columbian College of Arts & Sciences, George Washington University, Washington, D.C., United States of America; Fundaçao Oswaldo Cruz, BRAZIL

## Abstract

**Background:**

In the Indian sub-continent, visceral leishmaniasis (VL), also known as kala azar, is a fatal form of leishmaniasis caused by the kinetoplastid parasite *Leishmania donovani* and transmitted by the sand fly *Phlebotomus argentipes*. VL is prevalent in northeast India where it is believed to have an exclusive anthroponotic transmission cycle. There are four distinct cohorts of *L*. *donovani* exposed individuals who can potentially serve as infection reservoirs: patients with active disease, cured VL cases, patients with post kala azar dermal leishmaniasis (PKDL), and asymptomatic individuals. The relative contribution of each group to sustaining the transmission cycle of VL is not known.

**Methodology/Principal Findings:**

To answer this critical epidemiological question, we have addressed the feasibility of an approach that would use forensic DNA methods to recover human DNA profiles from the blood meals of infected sand flies that would then be matched to reference DNA sampled from individuals living or working in the vicinity of the sand fly collections. We found that the ability to obtain readable human DNA fingerprints from sand flies depended entirely on the size of the blood meal and the kinetics of its digestion. Useable profiles were obtained from most flies within the first 24 hours post blood meal (PBM), with a sharp decline at 48 hours and no readable profiles at 72 hours. This early time frame necessitated development of a sensitive, nested-PCR method compatible with detecting *L*. *donovani* within a fresh, 24 hours blood meal in flies fed on infected hamsters.

**Conclusion/Significance:**

Our findings establish the feasibility of the forensic DNA method to directly trace the human source of an infected blood meal, with constraints imposed by the requirement that the flies be recovered for analysis within 24 hours of their infective feed.

## Author Summary

Visceral leishmaniasis, also known as kala-azar, is a fatal form of leishmaniasis that is caused by the protozoan parasite *Leishmania donovani*. In the Indian sub-continent, the parasite is transmitted between people by the sand fly *Phlebotomus argentipes*. There are four different groups of infected people that can carry the parasite: active cases, cured cases, cases of post-kala azar dermal leishmanisis, and people with asymptomatic infections. The relative contribution of these different groups to sustaining the transmission cycle is not known. In the current work, we explore the feasibility of forensic DNA methods that could be applied to directly trace the human source of an infected blood meal in flies collected from high transmission areas. We report that a readable human DNA profile can be generated from the majority of sand flies so long as the flies are sampled within 24 hours after blood feeding. We developed a highly sensitive nested-PCR method that was able to detect parasites present in a fresh, 24 hour blood meal after feeding on an infected host.

## Introduction

Leishmaniasis is a disease caused by the parasitic protozoan *Leishmania*. The disease is transmitted by the bite of the female *Phlebotomine* sand fly that requires a blood meal for egg production. Visceral leishmaniasis (VL), also known as “kala-azar”, is the fatal form of Leishmaniasis that is characterized by bouts of fever, weight loss, enlargement of the spleen and liver and anemia. Approximately 300,000 new cases occur annually, with mortality rates of about ten percent (WHO, http://www.who.int/leishmaniasis/en). VL is caused by two closely related *Leishmania* species; *L*. *infantum* in the Mediterranean basin, North Africa and Central and South America, and *L*. *donovani* in the Indian subcontinent and east Africa. According to WHO, in India alone, around 10,000 cases were reported in 2014. Given the high rate of non-reported cases, the actual number is estimated to be around 100,000 (http://www.who.int/neglected_diseases/news/SEARO_poised_to_defeat_VL/en/), the majority of whom live in poor, rural settings in the northeast state of Bihar [1].

In northeast India, VL is transmitted by *Phlebotomus argentipes* [2]. While the infection reservoirs for VL in Europe, North Africa, and Brazil are animals, mainly dogs [3, 4], in the Indian subcontinent the disease is considered to have an anthroponotic transmission cycle, and no non-human reservoirs have been confirmed. Nonetheless, the relative importance of the different infected human populations in sustaining the transmission cycle is not known. There are four distinct groups of *L*. *donovani*-exposed human subjects that can serve as potential reservoirs for VL: active patients, cured cases, asymptomatic individuals and patients with post-kala azar dermal leishmaniasis (PKDL). PKDL is a complication of VL characterized by the appearance of skin nodules post-VL treatment. PKDL patients were thought to be the main reservoir for kala azar as their skin nodules contain large numbers of parasites that can be picked up by the vector [5]. As the low prevalence of PKDL cases in India, approximately 15% of VL patients [6], is unlikely to maintain the intensity of transmission observed, the potential contribution of asymptomatic cases has also been considered. Incident asymptomatic infections are far more frequent than incident disease, ranging from 6.1:1 to 17.1:1 in high-endemic villages of India and Nepal [7]. At least in the case of canine VL, it is known that asymptomatic infections can be highly transmissible to vector sand flies [8], and mathematical modeling suggests a major role of asymptomatics in driving transmission of human VL in India [9].

Identifying the human-subjects groups serving as infection reservoirs may be possible via xenodiagnostic studies using safe, laboratory-reared colonies of vector sand flies. Early studies employing direct xenodiagnosis of human VL patients were critical in helping to establish *P*. *argentipes* as the natural vector of *L*. *donovani* transmission in India [10]. And as mentioned, sand fly infections following exposure of the flies to nodular PKDL lesions has been accomplished on two occasions [5, 11]. Extending xenodiagnostic studies to include human subjects from across the infection spectrum would establish the relative potential of each exposure group to maintain the transmission cycle. However, this approach does not directly identify the source of an infected blood meal in sand flies captured from a transmission focus. In addition, xenodiagnostic studies require substantial infrastructures to establish and maintain a large working colony of *P*. *argentipes* that is safe to feed on human subjects, and the willingness of human volunteers to submit to this protocol. Another way to address this question is by using forensic DNA methods. This approach would involve recovering human DNA from blood-fed *L*. *donovani*-infected flies captured in high VL transmission areas. The DNA profiles would then be matched to reference samples taken from infected individuals in the same areas.

In the forensic community, the most commonly used method for human identification is the analysis of highly polymorphic Short Tandem Repeat (STR) markers. Forensic STR kits presently available have been developed to enable the generation of DNA profiles from as little as 100 pico grams of DNA. These kits can type multiple highly polymorphic STRs in a single reaction, producing profiles that are unique in a population and that can be matched to a reference database for source identification. Recovering human DNA and obtaining interpretable STR profiles from blood-sucking insects has been successfully demonstrated in mosquitos and has been used in epidemiologic studies. STR identification was used to determine the feeding preferences of *Aedes aegyptii*, the mosquito vector of the dengue virus, thus demonstrating the role played by a migrating population in spreading the virus [12, 13]. A more recent study determined that *Culicinae* mosquitos can be relevant to a criminal investigation when present at a crime scene, as human DNA can be successfully typed from these insects 56 hours after a blood meal has been taken [14].

The application of a similar approach to sand flies has not been previously reported, and could be challenging due to the fact that the blood meals of these insects are smaller and their digestion is faster compared to mosquitos. The volume of a fully engorged sand fly like *P*. *argentipes* is 0.63–0.73μl [15] whereas the volumes of blood meals in an engorged mosquito vary between 2–3 μl and remain >1 μl even 48 hours post blood meal (PBM) [16, 17]. The objective of the current study was to determine the feasibility of using a forensic DNA approach to identify the human source of a sand fly blood meal in a colonized population of *P*. *argentipes*. We conclude that a useful DNA fingerprint can be obtained within the first 24 hours of blood feeding, and that this time frame is compatible with detection of *L*. *donovani* in sand flies engorged on an infected host.

## Materials and Methods

### Ethics statement

The Office of Human Subjects Research Protections came to a determination of 'Excluded from IRB Review' per the requirements of 45 CFR 46 and NIH policy to obtain sand flies with human blood for the project, ‘Application of forensic DNA methods to sand fly blood meal analysis’. Exempt #: 13124. All hamster studies were carried out in strict accordance with the recommendations in the Guide for the Care and Use of Laboratory Animals of the National Institutes of Health. The protocol was approved by the Animal Care and Use Committee of the NIAID, NIH (protocol number LPD 68E). For anesthesia and sedation of hamsters, we used Telazol (100 mg/ml stock) and xylazine (20 mg/ml stock) given IP at a dose of 50 mg/kg Telazol and 5 mg/kg xylazine.

### Sand fly colonies and *Leishmania* parasites

The laboratory colony of *P*. *argentipes* originated from Aurangabad in Maharashtra state in India and was established and maintained as a colony at the Department of Entomology in Walter Reed, Army Institute of Research. The *L*. *donovani* Indian strain Mongi (MHOM/IN/83/Mongi-142) was used in this study. The parasites were passed in hamsters and grown as promastigotes in Medium199 as described elsewhere [18]. All hamster studies were carried out in strict accordance with the recommendations in the Guide for the Care and Use of Laboratory Animals of the National Institutes of Health. The protocol was approved by the Animal Care and Use Committee of the NIAID, NIH (protocol number LPD 68E). All hamsters were maintained at the NIAID animal care facility under specific pathogen-free conditions.

### Sand fly feeding on human volunteers and infected hamsters

Two adult human volunteers were used for this study, which, under a determination by The Office of Human Subjects Research Protections, NIH, was excluded from IRB review. To obtain human DNA profiles from blood fed flies, approximately 50, two to six-days-old *Phlebotomus argentipes* females were placed in a cylinder of clear polycarbonate plastic (1.5 inches high and 2 inches in diameter) closed at one end with a polycarbonate disk. The open end of the cup was covered with a piece of fine-mesh and held in place with an O-ring. The cylinder was attached to the leg of two subjects (D and P) with the open end facing their skin. Flies were allowed to feed for one hour at room temperature. Blood fed female were kept in 26°c incubators and sampled immediately, one, two, three and five days post feeding (T-0,1,2,3 and 5 respectively). After CO_2_ anesthetization, each fly was put into an individual Eppendorf tube and frozen at -80°C until processed.

For the detection of *L*. *donovani* in flies fed on infected hamsters, 5x10^7^ ficoll-purified *Leishmania donovani* metacyclics were intravenously injected into Syrian Golden hamsters. After approximately two months when the hamsters had lost around 30% of their weight, they were anesthetized, their abdomens were shaved, and they were exposed to 100–150 *P*. *argentipes* flies for approximately one hour at 26°C in the dark. Non-fed females were removed from the cage and blood-fed flies were CO_2_ anesthetized and immediately processed for DNA analysis. Some flies were maintained alive for microscopic evaluation of their infections at later time points.

### Preparation of DNA

Whole flies were put in 1.5 mL Eppendorf tubes and DNA was extracted using a QiAamp DNA investigator kit (Qiagen # 56504: reference protocol on hair and fingernail clips extraction). Flies were incubated at 56°C for one hour on a thermomixer (900RPM), then homogenized with pestles, followed by incubation for an additional hour at 56°C. The elution was passed a second time through the column for maximal DNA recovery. Final elution volume was 30 μl. DNA yields and concentrations are summarized in Table 1.

**Table 1:** Total and human DNA extracted from the flies.

Source/Time point	Subject	Fly	Total DNA (ng)	Human DNA (ng)	Human DNA used for amplification	Profile
Buccal swabs	D		1200	866.668	288.889	++
	P		600	356.719	118.906	++
		a	444	1.892	0.631	++
	D	b	360	4.278	1.426	++
		c	891	2.102	0.701	++
		d	717	1.442	0.481	++
Sand fly T-0 full		a	432	0.323	0.108	-
	P	b	297	0.688	0.229	+
		c	252	0.376	0.125	++
		d	549	0.873	0.291	+
		Average	492.75	1.497	0.499	
		Stdv	217.8	1.306	0.435	
		a	288	0.091	0.030	-
	D	b	549	0.000	0.000	-
		c	804	0.505	0.168	+
		d	870	1.654	0.551	++
Sand fly T-0 partial		a	258	0.131	0.044	+
	P	b	390	1.683	0.561	++
		c	261	0.000	0.000	-
		d	441	0.456	0.152	+
		Average	482.6	0.565	0.188	
		Stdv	240.7	0.707	0.236	
		a	292	0.009	0.003	+
	D	b	290	0.032	0.011	-
		c	284	0.005	0.002	+
		d	626	0.094	0.031	+
Sand fly T-1		a	482	0.103	0.034	+
	P	b	538	0.000	0.000	-
		c	376	0.010	0.003	-
		d	328	0.189	0.063	+
		Average	402	0.055	0.018	
		Stdv	130.8	0.068	0.023	
		a	340	0.000	0.000	-
	D	b	360	0.000	0.000	-
		c	282	0.006	0.002	-
		d	286	0.010	0.003	-
Sand fly T-2		a	340	0.000	0.000	-
	P	b	280	0.014	0.005	-
		c	360	0.050	0.017	-
		d	260	0.021	0.007	+
		Average	313.5	0.013	0.004	
		Stdv	40.5	0.017	0.006	
		a	490	0.004	0.001	-
	D	b	258	0.000	0.000	-
		c	400	0.000	0.000	-
		d	396	0.000	0.000	-
Sand fly T-3		a	416	0.000	0.000	-
	P	b	652	0.000	0.000	-
		c	218	0.000	0.000	-
		d	272	0.000	0.000	-
		Average	387.75	0.001	0.000	
		Stdv	141.6	0.001	0.000	
		a	1077	0.000	0.000	-
	D	b	333	0.000	0.000	-
		*c	891	0.074	0.025	-
		d	582	0.000	0.000	-
Sand flyT-5		a	417	0.000	0.000	-
	P	b	270	0.000	0.000	-
		c	1026	0.000	0.000	-
		d	ND	0.000	0.000	-
		Average	656.6	0.009	0.003	
		Stdv	338.0	0.026	0.009	

Reference DNA was extracted from buccal swab sampled from two individuals, D and P. Flies with full and partial blood meals were collected immediately after the feeding (T-0) and after 1,2 and 3 (T-1, T-2 & T-3) days PBM. Four flies fed on each individual were analyzed at each time point. The average and standard deviation refers to all eight samples from each sampling time point. All samples were eluted in 30 μl and 10 μl were loaded in the PCR reactions.

(++) Full profile, both alleles of heterozygous loci are readable, or in homozygous loci the single peak is above the stochastic threshold (ST) which represents the relative fluorescent unit (RFU) value above which there is very low probability that a second allele in a truly heterozygous locus has dropped out.

(+) Partial profile, only one allele of heterozygous loci is readable or in homozygous loci the RFU value of the allele is below ST.

(-) No profile, none of the alleles were detectable.

(*) Human DNA detected is from a source of contamination because the STR profile did not match either of the reference DNA profiles.

Reference DNA samples of the two subjects on whom the flies fed were collected using buccal swabs. DNA from the reference swabs was extracted using the EZ1 DNA Investigator Kit (Qiagen– 952034) on a BioRobot EZ1 [19]. Total DNA from all samples was quantified with a NanoDrop 1000 (Thermo Scientific).

### PCR amplification of Leishmania and human DNA; DNA fingerprint analysis

For the detection of *L*. *donovani* in blood-fed *P*. *argentipes* and specifically in flies with fresh blood meals, a “nested PCR” approach was used. The target gene was NADH dehydrogenase subunit 5 (ND5), which is located on the maxicircle DNA [20] and previously used for *Leishmania* genotyping. The primer sequences for the external PCR round [18] were: Fwd, 5’-GAYGCDATGGAAGGACCDAT-3’ and Rev, 5’-CCACAYAAAAAYCAYAANGAACA-3’. The PCR protocol was 25 Cycles of 94°C 30 sec, 60°C 30 sec and 72°C for 30 sec. The PCR products were purified in “Wizard SV Gel and PCR cleanup system (Promega A11-20)” and were used as templates for a second internal PCR. The primer sequences specifically designed for this work were: Fwd, 5’-ATACATGCAGCAACCTTAGTTG-3’ and Rev, 5’ CATATTGTACTAAATGCAACATACC-3’. The PCR protocol was 35 cycles of 94°C 30 sec, 62°C 30 sec and 72°C for 20 sec.

Human DNA was quantified with Real Time PCR using Applied Biosystems’ Quantifiler Human DNA Quantification Kit (#4482911) on an ABI 7900 Real Time PCR machine according to the manufacturer’s instructions. Ten μl of *P*. *argentipes* DNA extract and ~ 250 pgrams of reference DNA were used as a template for STR amplification using AmpFlSTR Identifiler Plus (Applied Biosystems 4427368) following the manufacturer’s instruction. Capillary Electrophoresis of the PCR products was performed on an ABI PRISM 3130 Genetic Analyzer, and profiles were analyzed with GeneMarkerHID 1.9 software. Electropherogram interpretation was performed mimicking standard operating procedures followed in forensic DNA analysis [21], taking into account only data that is above the analytical threshold (AT), which defines the minimum height requirement, expressed in relative fluorescent units (RFU) above which detected peaks can be reliably distinguished from background noise. The AT is empirically determined by an internal validation and peaks below this level are not considered reliable and not used for interpretation. Loci without peaks above the AT were not used for a comparison with the reference data. The random match probabilities (RMP) (or chance of a coincidental match) of the complete and partial STR profiles obtained were calculated using population allele frequencies published in the Identifiler Plus user manual.

## Results

### Quantity of human DNA in sand flies is proportional to blood meal size

**Fig 1:**
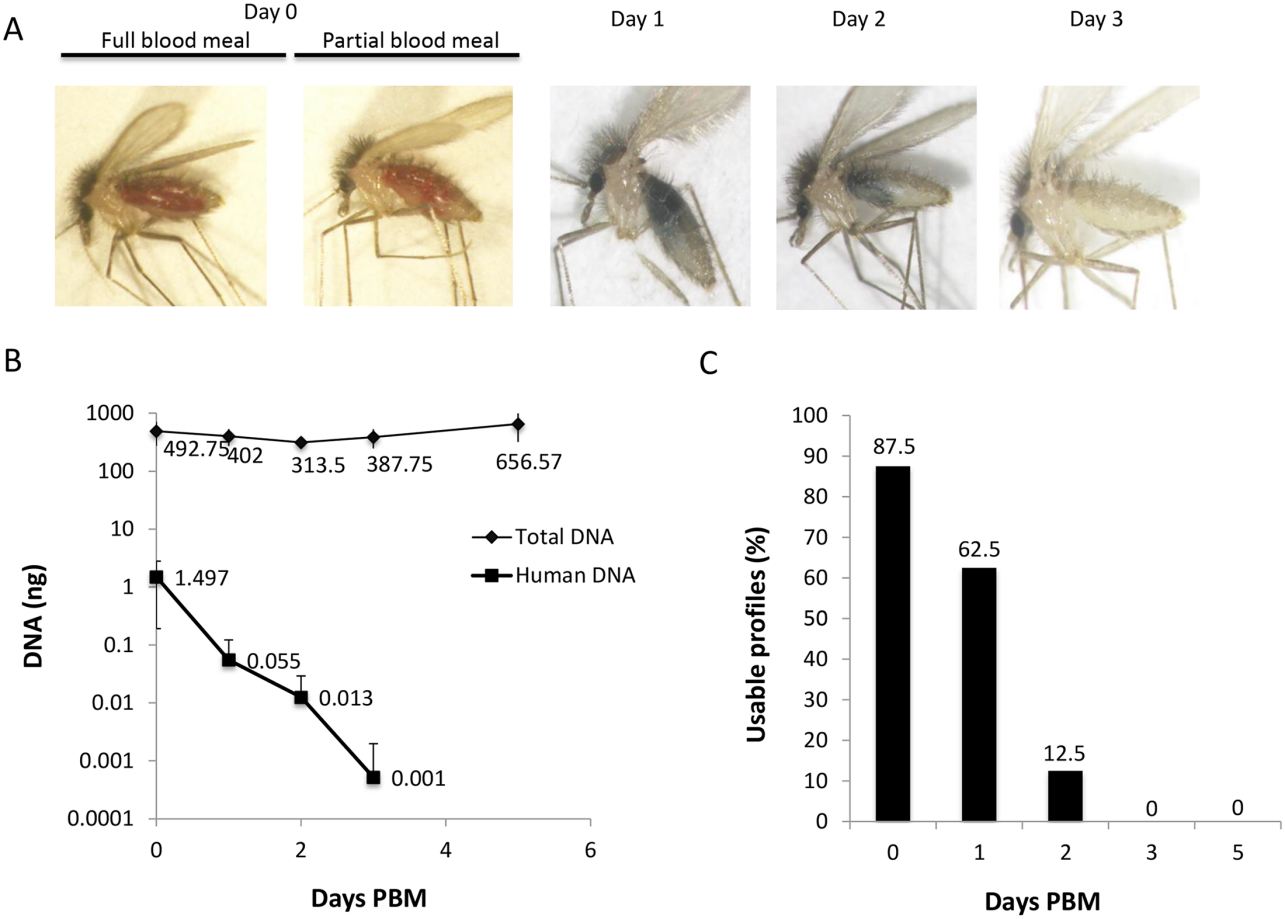
The kinetics of blood meal digestion determines the time frame for detecting human DNA. (A) Photomicrographs depicting flies with a full or partial blood meal at day 0, and the kinetics of blood meal digestion on days 1–3 PBM. (B) Total and human DNA at days 0, 1, 2, 3 and 5 PBM. The results indicate the mean value of eight different flies, four from each subject ±SD. (C) The percentage of flies that yielded a STR profile suitable for comparison out of eight flies tested on each day.

Eight *P*. *argentipes* flies were processed at each time point, four from each of the populations fed on the two human volunteers. The amount of total DNA that was extracted from the flies was relatively stable throughout the Experiment (Fig 1B and Table 1). On day zero (T-0), total DNA was 492.75 ± 217.8 ng and 482.6 ± 240.7 ng for flies with full and partial blood-meals respectively. On the following days post feeding, total DNA amounts were 402± 130.8, 313.50 ± 40.5, 387.75 ± 141.6 and 656.57 ± 338 ng, one, two, three and five (days T-1, 2, 3 and 5) post feeding respectively (Fig 1B and Table 1). On day zero, the amount of human DNA in fully engorged flies was more than three orders of magnitude lower than the total DNA from the whole fly, and the human DNA rapidly declined with each following day. The decline in human DNA was correlated with the decline in the blood meal size (Fig 1A and 1B). Fully engorged flies at T-0 yielded an average of 1.5±1.3 ng human DNA. Interestingly, flies that fed on subject D had bigger blood meals then those fed on subject P, and this correlated directly with the amount of human DNA that was recovered; 2.4±1.3 and 0.6±0.2 ng human DNA from individuals D and P respectively (Table 1, T-0 full blood meals). We did not observe differences in blood meal size at T-0 between flies that were partially fed on the two individuals, nor between these groups of flies in the amounts of blood remaining in the following days. The average human DNA amounts decreased roughly ten-fold with each day PBM. Flies collected one and two days after the blood meal yielded 0.055±0.07 and 0.013±0.02 ng human DNA respectively (Fig 1B and Table 1). Three days post feeding, the flies had completely digested the blood meals (Fig 1A), and only one fly from this time point yielded a detectable amount of human DNA (0.004ng). Human DNA was not detected five days post feeding in any of the flies.

### Obtaining human DNA profiles is constrained to 0–48 hours PBM

One third of the DNA extraction volume was used for the multiplex amplification of STR markers. An average of 0.49±0.43 ng DNA from fully engorged flies on day 0 were used for PCR. That amount enabled 7/8 (87.5%) quality profiles (Fig 1C; Table 2). PCR amplification of 0.48–1.43 ng human DNA from subject D yielded full STR profiles in all 4 T-0, fully engorged samples, generating a RMP of one in 2.4x10^21^ (Table 2). The RMP of a sample represents the overall chance for a random unrelated person to have the same genotype in the population and it is calculated based on allele frequencies. Only 0.1 to 0.29 ng of DNA from subject P was used for amplification, which resulted in three usable profiles out of four flies. Two were partial profiles yielding a RMP of 1 in 1.6x10^12^ and 1 in 1.3x10^20^ for fly b and d respectively. The third profile was full with a RMP of 1 in 6x10^20^ (Tables 1 and 2).

**Table 2:** Allele frequency calculations in flies yielding usable profiles.

Locus	T-0 full blood meal							T-0 partial blood meal					T-1					T-2
	D				P			D		P			D			P		P
	a	b	c	d	b	c	d	c	d	a	b	d	a	c	d	a	d	d
D8S1179	+/+	+/+	+/+	+/+	+/+	+/+	+/+	+/+	+/+	+/-	+/+	+/+	+/+	+/+	+/+	+/+	+/+	-/-
D21S11	+/+	+/+	+/+	+/+	-/-	+/+	+/+	-/-	+/+	+/-	+/+	+/+	-/-	+/+	+/-	+/+	+/+	+/-
D7S820	+/+	+/+	+/+	+/+	-/-	+/+	+/+	-/-	+/+	+/-	+/+	+/-	+/-	+/+	+/-	+/+	+/+	-/-
CSF1PO	+/+	+/+	+/+	+/+	-/-	+/+	+/-	-/-	+/+	+/-	+/+	+/-	-/-	+/+	+/-	+/+	+/+	+/-
D3S1358	+/+	+/+	+/+	+/+	+/+	+/+	+/+	+/-	+/+	+/-	+/+	+/+	+/-	+/+	+/-	+/+	+/+	+/-
THO1	+/+	+/+	+/+	+/+	+/+	+/+	+/+	-/-	+/+	+/-	+/+	+/-	+/+	+/+	+/+	+/+	+/+	+/+
D13S317	+/+	+/+	+/+	+/+	+/+	+/+	+/+	+/-	+/+	+/+	+/+	+/+	+/+	+/+	+/+	+/-	+/+	+/+
D16S539	+/+	+/+	+/+	+/+	-/-	+/+	+/+	-/-	+/+	+/+	+/+	+/-	+/+	+/+	+/+	+/+	+/+	+/-
D2S1338	+/+	+/+	+/+	+/+	+/+	+/+	+/+	-/-	+/+	+/-	+/+	+/+	+/-	+/—	+/-	+/-	+/+	+/+
D19S433	+/+	+/+	+/+	+/+	+/+	+/+	+/+	+/+	+/+	+/-	+/+	+/+	+/-	+/-	+/+	+/+	+/+	+/+
vWA	+/+	+/+	+/+	+/+	-/-	+/+	+/+	+/-	+/+	+/+	+/+	+/+	+/-	+/+	+/+	+/+	+/+	+/+
TPOX	+/+	+/+	+/+	+/+	+/+	+/+	+/+	+/-	+/+	+/+	+/+	+/-	+/-	+/+	+/-	+/-	+/+	+/-
D18S51	+/+	+/+	+/+	+/+	-/+	+/+	+/+	+/-	+/+	-/-	+/+	+/-	+/-	+/+	+/-	+/-	+/+	+/-
D5S818	+/+	+/+	+/+	+/+	-/+	+/+	+/+	-/-	+/+	+/+	+/+	+/+	-/-	+/+	+/+	+/+	+/+	+/-
FGA	+/+	+/+	+/+	+/+	+/+	+/+	+/+	+/+	+/+	+/+	+/+	+/-	+/+	+/+	+/+	+/-	+/+	+/+
^Δ^Total	15	15	15	15	10	15	15	7	15	15	15	15	12	15	15	15	15	13
* RMP, 1 in	2.4E+21	2.0E +21	2.4E +21	2.4E + 21	1.6E + 12	6.2E + 20	1.3E + 20	1.3E +11	2.4E +21	1.1E +13	6.2E +20	1.8E +14	3.4E +10	6.2E +18	1.8E +15	1.1E +16	6.2E +20	3.5E +12

(D and P) source of human blood meals; (a-d) individual flies with usable STR profiles;

(++) both alleles of heterozygous loci were readable, or in homozygous loci the single peak was above stochastic threshold (ST);

(+/-) only one allele of heterozygous loci was readable or in homozygous loci the RFU value of the allele was below ST;

(-/-) both alleles were not detectable;

(Δ) total number of markers of which at least one allele was readable;

(*) RMP was calculated for each sample based on population specific allele frequencies.

For samples collected the following day (T-1), an average of 0.018±0.0023 ng human DNA was used for PCR. Still, 5/8 (62.5%) flies produced sufficient human DNA profiles. Three flies fed on subject D, from which 0.003, 0.002 and 0.031 ng of human DNA were used for PCR, produced partial profiles with RMPs of 1 in 3.4x10^10^, 1 in 6.2x10^18^ and 1 in 1.8x10^15^, respectively. From subject P, 0.034 and 0.063 ng human DNA from two flies yielded partial and full profiles with RMPs of 1 in 1.1x10^16^ and 1 in 6.2x10^20^, respectively. The rest of the samples from this time point did not have sufficient human DNA to generate profiles suitable for comparison. For flies sampled at T-2, only one fed on subject P had enough human DNA to produce an STR profile. Using 0.007 ng of this DNA, a partial profile was generated which still gave a RMP of 1 in 3.5x10^12^.

Three days post feeding, the flies had completely digested the blood meals (Fig 1A). Only one fly from this time point yielded detectable amounts of human DNA (0.0003 ng), which was too low to produce a STR profile suitable for comparison (Table 1). No human DNA was found in any of the flies collected 5 days PBM.

Altogether, the results show that above 0.5 ng of human DNA, high quality, full STR profiles can be obtained. This correlates with fully engorged flies sampled on the day of the bite. The presence of a partial blood meal, due either to incomplete engorgement or to blood meal digestion, and yielding between 0.004 and 0.5 ng human DNA, can generate partial STR profiles that are still sufficient for human identification. Results summarized in Table 1 suggest that the sensitivity of the assay is down to the single cell range, since the amount of DNA in a human cell is 0.006 ng. Thus, the first twenty-four hours PBM is the time frame in which human DNA profiles can be generated from most *P*. *argentipes* sand flies.

We have also determined the effect of storage conditions on the integrity of human DNA that can be obtained from the flies. Flies were fed on a human blood meal source and collected immediately after the feeding (T-0) and 1 day PBM (T-1). At each time point, 12 flies were put in 96% ethanol, half of the flies were placed in -80°C and the other half were stored at 4°C for five days. The average of total human DNA obtained from flies collected at T-0 was 7.63±1.46 and 7.27±5.64 for flies kept in -80°C and 4°C, respectively; at T-1the total human DNA amounts were 3.71±3.32 and 4.37±3.81, respectively (Table 3). Thus, storage under conditions more applicable to the availability of resources in the field did not compromise human DNA integrity.

**Table 3:** The effect of storage conditions on the integrity of human DNA.

		Total Human DNA (ng)	
Time point	Fly	* Stored in -80°C	^Δ^ Stored in 4°C
T-0	a	8.86	5.67
	b	6.97	4.62
	c	6.22	18.69
	d	6.83	4.41
	e	6.93	4.21
	f	9.98	6.04
	Average	7.63	7.27
	Stdv	1.46	5.64
T-1	a	9.68	0.59
	b	3.19	11.58
	c	5.32	3.04
	d	1.41	2.68
	e	1.38	3.28
	f	1.30	5.03
	Average	3.71	4.37
	Stdv	3.32	3.81

*Flies were put in absolute ethanol at -80°C freezer immediately after collection.

^Δ^ Flies were kept in 96% ethanol at 4°C for five days.

### Detection of *L*. *donovani* in fresh blood meals from *P*. *argentipes*

**Fig 2:**
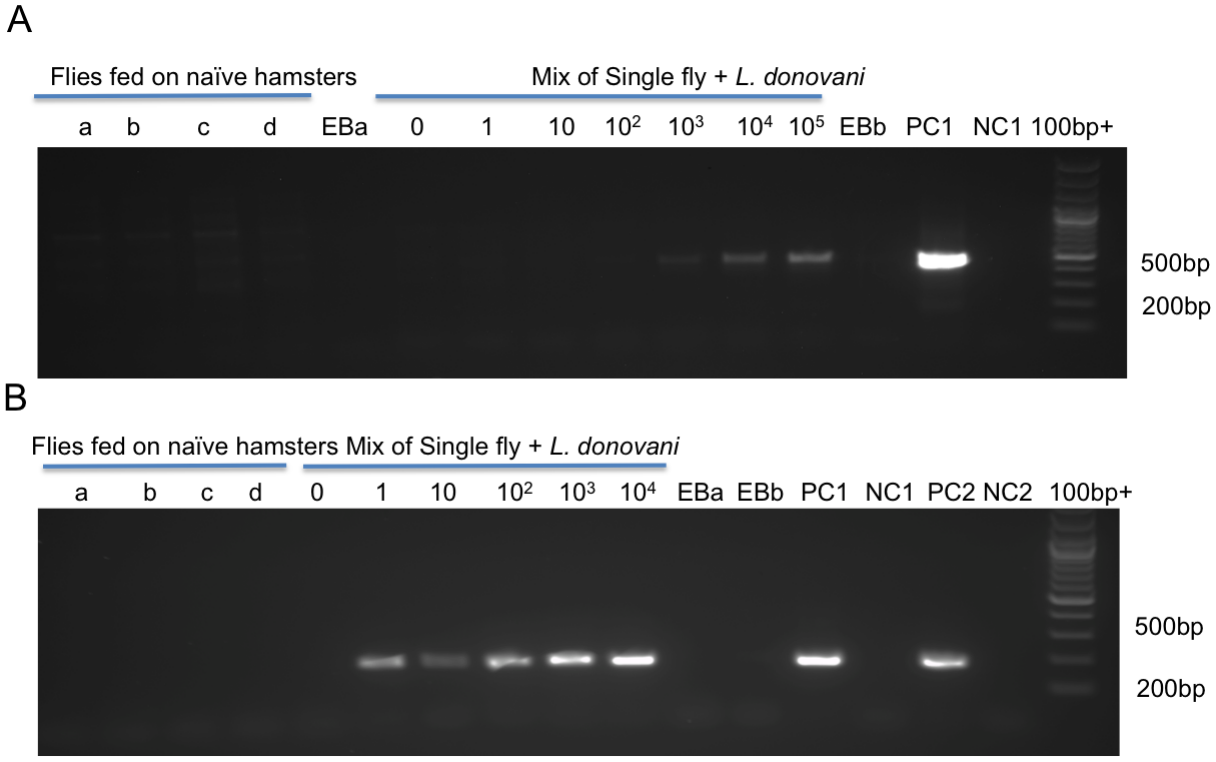
ND5 PCR for detection of different levels of *L*. *donovani* in *P*. *argentipes* flies. Standard direct PCR (A) or nested PCR (B) reactions on DNA extracted from individual blood fed flies fed on hamsters and mixed with the indicated number of parasites. A-d, flies fed on naïve hamsters as control for primer specificity. EBa, extraction buffer blank for DNA extracted from a-d flies. EBb, extraction blank for DNA extracted from the mixture of flies and parasites. PC1 and NC1 are positive and negative controls for the standard ND5 PCR (A) and the external PCR in the nested reaction (B). Both PC1 and NC1 from the external reaction were cleaned and used as templates for the internal PCR. PC2 and NC2 are positive and negative controls for the second internal nested PCR (B).

The findings discussed above indicate that flies must be collected within the first 24 hours after the blood meal if human DNA profiles are to be reliably obtained. It was therefore important to determine whether the number of parasites present in a fresh blood meal obtained from a potentially infectious reservoir host, and prior to their expansion as promastigotes, is above the threshold detection limits of the PCR used to identify infected flies. When tested on serial dilutions of cultured *L*. *donovani* promastigotes mixed with a single *P*. *argentipes* fly, ND5-targeted PCR resulted in specific amplification with no false positives. However, significant amplification was observed only in DNA from flies mixed with 10^3^ or a greater number of parasites (Fig 2A). To increase the sensitivity of the assay, a nested PCR approach was developed that allowed ND5 gene detection from flies mixed with as few as 1 parasite (Fig 2B).

**Fig 3:**
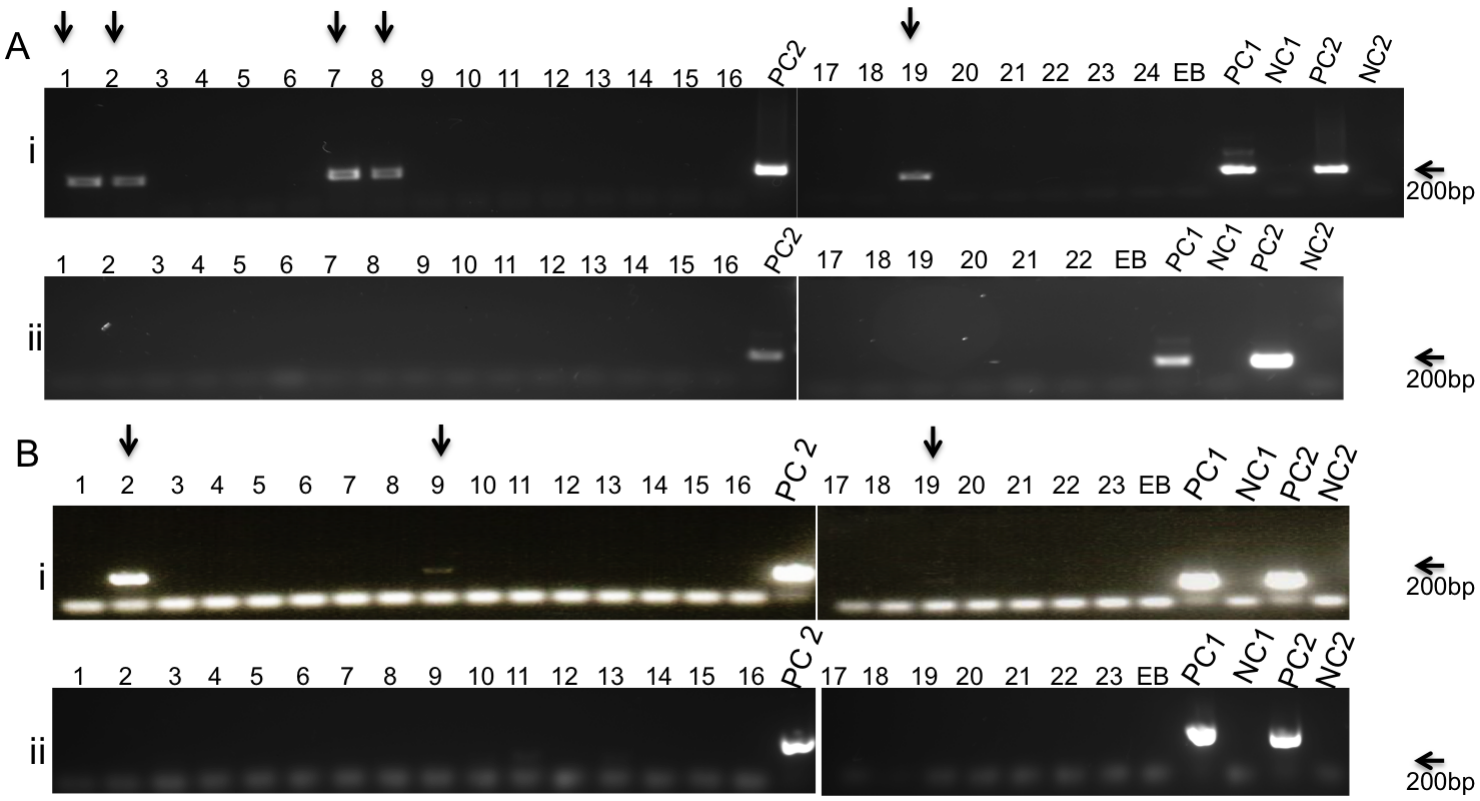
Detection of *L*. *donovani* amastigotes in a fresh blood meal. Two independent experiments (A and B) showing nested PCR amplification of ND5 gene from flies fed on infected (i) or naïve (ii) hamsters. Arrows indicate flies showing positive reaction. EB, DNA extraction blank; PC, positive control for the first, external PCR (PC1) or the second internal fragment (PC2). NC, negative control for the first, external (NC1) or the second, internal (NC2) PCR reactions. The faint bands observed above the primers at lanes 11 and 13 in panel B ii are not at the same size of the true PCR product and are most likely a product of primer dimerization.

The ND5 nested-PCR assay was tested in two separate experiments (Fig 3A and 3B) on flies collected at the same day of the feeding (T-0). A total of ninety-two flies were tested, 47 of them fed on a *L*. *donovani* infected hamster and the remaining fed on a naïve hamster as control. No PCR amplifications were detected from the control flies, in both the external and nested amplifications. Out of the 47 flies fed on the infected the hamster, 8 yielded positive PCR amplification for the ND5 target, 5 in the first experiment and 3 in the second experiment. To further evaluate the reliability of the detection method, infections were allowed to develop in a portion of the flies. When flies were collected 9–11 days PBM and their dissected midguts examined microscopically, parasites were observed only in 1 out of 9 and 1 out of 6 flies in experiments A and B, respectively, consistent with the low frequency of infected flies determined by the nested-PCR approach.

## Discussion

This study both confirms the feasibility of applying forensic DNA methods to epidemiological studies of human VL and points out some of the limitations of this approach. It is clear that the ability to obtain human DNA profiles depends on the size of the original blood meal and the kinetics of its digestion (or loss). We have shown the minimum amount of human DNA that is needed for the amplification of a reliable human profile and provided a time frame in which this amount of DNA can be obtained from blood-fed *P*. *argentipes* flies. The data show that in the first 24 hours PBM the flies carry enough blood to obtain a human profile suitable for comparison. It becomes significantly less likely to obtain comparable profiles from flies analyzed at 48 hours or longer after the bite. Although the role of secreted nucleases by midgut cells cannot be ruled out, the reduction in the efficiency of generating STR profiles each day PBM is more likely attributed to the excretion of the blood meal remnants from the gut. By comparison, STR human DNA profiles were recovered from all *Culicinae* mosquitos 48 hours PBM, and from 62% and 27% of the mosquitos at 56 and 72 hours PBM [14], indicating their slower rate of blood digestion and loss, and suggesting that DNA in the blood meals of blood sucking diptera is not completely degraded by nucleases even several days after the feeding.

Should a fly feed off of two different individuals prior to being captured, the assay would yield a mixed profile. The interpretation of mixed profiles is more challenging and generally less informative than single source samples. Yet a mixed profile can still be useful for human identification, depending on the amount of DNA and the ratio between the two contributors, both subjects could be identified. The interpretation should be conducted following forensic STR interpretation procedures commonly used by practitioners (https://www.fbi.gov/about-us/lab/biometric-analysis/codis/swgdam-interpretation-guidelines).

The limited time frame in which flies can be used for human profile amplification raises a number of challenges in conducting an epidemiological study that attempts to determine the reservoir for anthroponotic VL. Studies conducted in high transmission areas in northeast India showed that among a random collection of 1397 *P*. *argentipes* flies, only 4 flies (0.28%) were both blood engorged and infected [22]. Moreover, as the age of the blood meals in those studies was not reported, the number of flies that would have been useful for DNA fingerprinting might have been lower. These findings and the present study suggest that in order to perform a successful epidemiological study that employs DNA fingerprinting, the sampling would need to be conducted on a much larger scale.

Another issue associated with the short time frame in which STR profiling is effective is the potentially low number of the parasites present during the first 24 hours after the fly has acquired an infective blood meal, coinciding with only the very early stage of their transformation to and expansion as replicating promastigotes. To overcome this issue, a nested PCR targeting maxicircle kDNA was developed. A nested PCR targeting ribosomal DNA has been previously shown to substantially enhance the sensitivity and specificity of *Leishmania* detection in the skin compared to conventional techniques [23]. We chose to target maxicircle kDNA sequences because the copy number of the maxicircles is 20–50 per cell, which makes them more sensitive PCR amplification targets compared to chromosomal genes, though less sensitive than minicircle targets. On the other hand, as maxicircles are in much lower copy number than minicircles, contamination becomes less likely in environments routinely exposed to *Leishmania*. This observation concurs with Abbasi et al. [24], who reported inconsistencies and high rates of false positives when targeting the minicircles, particularly when applied to quantify parasite loads that were close to the detection threshold (1–10 parasites).

The low number (17%) of PCR-positive flies detected in flies fed on infected hamsters was surprising considering the symptoms that indicated an advanced stage of VL in those hamsters (30% weight loss). This may raise a question about the sensitivity of the detection method, especially when applied to a fresh blood meal. However, this observation was consistent with the low number of *L*. *donovani*-positive flies detected when infections were allowed to develop further. The inconsistency with which blood fed flies picked up infections might indicate that the parasites were not acquired from peripheral blood but from focalized concentrations of parasitized cells in the skin.

The final issue in the field application of this approach is the feasibility to obtain the appropriate reference DNA profiles so that the link between an infected fly with a readable STR profile and its human source can be made. Since sand flies are weak fliers and travel in short hops rather than in sustained flight, there is a high likelihood that their human blood meal source will be found living or working within close proximity to the location of the capture. While the infection histories of these individuals would no doubt also be obtained, their direct link to an infected blood meal could only be made using the forensic approach. It is important to add that the key unanswered question in the epidemiology of VL in India is whether healthy individuals with asymptomatic infections can transmit infection to the vector. There is still no uniformly accepted method to identify these individuals. For example, while a positive PCR for parasite DNA in peripheral blood is thought to provide the best evidence for sub-clinical infection, a negative PCR is meaningless for the purpose of identifying potential infection reservoirs if transmissions occur when flies pick up parasites in the skin. Lastly, based on the experience of the genome-wide association studies in which high quality genomic DNA was obtained from buccal swabs from over 2000 individuals in a high transmission area in Bihar [25], the selected sampling of individuals living or working close to the site of infected fly capture seems an achievable undertaking, and any ethical concerns can be fully met.

In conclusion, this study demonstrates for the first time that the use of forensic DNA methods enables identification of the human source of a sand fly blood meal, and may therefore be used to directly trace the source of an infected blood meal in flies recovered from kala-azar endemic zones. Understanding the dynamics and epidemiology of anthroponotic transmission holds clear importance for the development of control strategies for human VL.
